# On Phloridzine and Its Uses

**Published:** 1863-11

**Authors:** D. Ricci


### ON PHLORIDZINE AND ITS USES.—By Dr. D. Ricci.

Phloridzine is a neutral principle existing in considerable quantities in the bark of the root of the apple, plum, and cherry trees, but principally in the root of the apple tree. It appears in the market in the form of a dirty-whitish powder, consisting of broken-up, silky needles, somewhat resembling quinine which has not been well bleached, and when rubbed between the fingers it has a soft, velvety feel, very like that of French chalk. When crystalized by slow cooling from a dilute solution, previously treated with freshly prepared animal charcoal, phloridzine may be obtained perfectly white, and in the form of long silk needles. Its taste is peculiar, being bitter at first, but afterwards somewhat sweetish, with a flavor of apples. Phloridzine differs from quinine by containing no nitrogen in its chemical composition, but in this respect it resembles salicine, to which it is much allied. Like salicine, it does not combine with acids to form salts, is very soluble in alcohol, ether, or boiling water, but requires one thousand parts of cold water for solution.

The cases in which Dr. De Ricci has employed phloridzine with most success have been certain forms of atonic dyspepsia occurring in delicate females, to whom it was impossible to administer either bark, quinine, or salicine in any shape, without bringing on serious nervous excitement. He has also found it extremely well adapted for the treatment of young children of delicate constitutional habit, or when recovering from whooping-cough, infantine fever, or any other disease. The doses he has employed are five grains three or four times a day for adults, and proportionately smaller doses for young children. In prescribing phloridzine it must be borne in mind that it is almost insoluble in cold water, but the addition of a very small quantity of ammonia instantly dissolves it; thus, by adding to an eight ounce mixture, containing a drachm of phloridzine, a few drachms of aromatic spirit of ammonia, the fluid which was previously milky becomes perfectly clear, and the addition of the aromatic spirit rather improves the mixture than otherwise. Dr. De Ricci relates the case of a young lady of a strumous constitution, suffering from chlorosis, in which the effects of phloridzine were manifestly favorable. The patient was unable to take iron in any shape, and both quinine and salicine equally disagreed with her; but phloridzine agreed perfectly well, and her constitution improved so much under its use that she was subsequently able to take citrate of iron and strychnia in grain doses, which ultimately effected a perfect cure. Dr. De Ricci thus recapitulates the advantages of this drug; it is tolerated in cases where neither quinine, nor salicine, nor bark, can be administered with impunity; it is particularly adapted to young children, it is not expensive, and it is abundantly supplied in Great Britain, thus rendering us independent of the rapidly diminishing cinchona forests of South America.—*Dublin Quar. Jour. of Medical Science, August*, 1863.

